# Molecular Glue cc‐885 Inhibits VHL‐Deficient Clear Cell Renal Cell Carcinoma via ETS1 Degradation

**DOI:** 10.1002/advs.202520237

**Published:** 2026-05-06

**Authors:** Taowei Yang, Qihao Li, Kun Ye, Kezhi Liu, Mi Zhou, Minyu Chen, Lican Liao, Kangbo Huang, Zhu Wang, Qiong Deng, Jieyan Wang, Meiyu Jin, Xu Chen, Hui Liang, Jiaxing Zhang, Junhang Luo

**Affiliations:** ^1^ Department of Urology The First Affiliated Hospital Sun Yat‐sen University Guangzhou Guangdong China; ^2^ Department of Oncology and Southwest Cancer Center Southwest Hospital of Third Military Medical University (Army Medical University) Chongqing China; ^3^ Department of Oncology The First Affiliated Hospital, Sun Yat‐Sen University Guangzhou Guangdong China; ^4^ Department of Urology Sun Yat‐sen University Cancer center Guangzhou Guangdong China; ^5^ State Key Laboratory of Oncology in South China Guangdong Provincial Clinical Research Center For Cancer Sun Yat‐sen University Cancer Center Guangzhou Guangdong China; ^6^ Department of Urology The People's Hospital of Longhua Shenzhen Guangdong China

**Keywords:** molecular cancer, molecular dynamics simulation, renal cell carcinoma, ubiquitination

## Abstract

Clear cell renal cell carcinoma (ccRCC) is a common and aggressive form of kidney cancer with VHL mutations present in > 50% of cases. These mutations lead to dysregulation of hypoxia‐inducible factors (HIFs) and activation of oncogenic pathways, making VHL‐deficient ccRCC a challenging target for therapy. In this study, we identified cc‐885, a molecular glue degrader, as a selective inhibitor of VHL‐deficient ccRCC. cc‐885 promotes ubiquitination and degradation of transcription factor ETS1, which cooperates with EPAS1 (HIF‐2α) to drive tumorigenesis. We demonstrated that cc‐885 selectively targeted the p51 and p42 isoforms of ETS1, disrupting p27/p51 balance and suppressing ETS1 transcriptional activity. Moreover, combining cc‐885 with an EPAS1 inhibitor, belzutifan, significantly enhanced the anti‐tumor efficacy. Our findings provide a novel and precise therapeutic strategy for VHL‐deficient ccRCC by targeting ETS1 degradation and disrupting the ETS1‐EPAS1 complex.

## Graphical abstract

VHL‐deficient kidney cancer lacks effective treatments. This study reveals that the molecular glue degrader cc‐885 hijacks the cellular recycling system to selectively destroy the oncogenic protein ETS1, effectively killing VHL‐mutant tumors. Combining CC‐885 with the approved drug belzutifan achieves powerful synergy, offering a promising new therapeutic strategy.

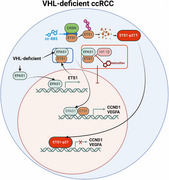

## 1 Introduction

Clear cell renal cell carcinoma (ccRCC) is the most prevalent form of kidney cancer, accounting for approximately 70% of all cases [1, 2]. The von Hippel‐Lindau (VHL) gene, a key tumor suppressor, is frequently mutated or deleted in ccRCC, leading to the accumulation of hypoxia‐inducible factors (HIFs), such as EPAS1 (HIF‐2α) [1, 3]. HIFs activate the transcription of oncogenic genes, including CCND1 and VEGFA, which drive tumor growth and angiogenesis [4, 5]. Although VEGF pathway inhibitors such as sunitinib are widely used in clinical practice, their efficacy is limited by drug resistance and toxicity [2, 6].

Targeted protein degradation (TPD) has emerged as a promising strategy for cancer therapy, particularly with the use of molecular glue degraders [7, 8, 9]. These small molecules act as “adhesives” to bridge E3 ubiquitin ligases and target proteins, leading to their ubiquitination and degradation by proteasomes [10, 11, 12]. However, the application of molecular glues in ccRCC, particularly in the context of VHL deficiency, remains unclear.

In this study, we identified and characterized cc‐885 as a selective molecular glue degrader of ETS1 in VHL‐deficient ccRCC. We demonstrated that cc‐885 induces the degradation of ETS1 through the Cereblon (CRBN) E3 ubiquitin ligase, disrupting the ETS1‐EPAS1 complex and suppressing oncogenic gene expression. Furthermore, we showed that combining cc‐885 with belzutifan, an EPAS1 inhibitor, resulted in a combined antitumor effect. Our findings provide a novel therapeutic approach for VHL‐deficient ccRCC and highlight the potential of molecular glues in modulating transcription factor networks.

## 2 Results

### 2.1 Selectivity of cc‐885 for VHL‐Deficient Renal Cancer Cells

**FIGURE 1:**
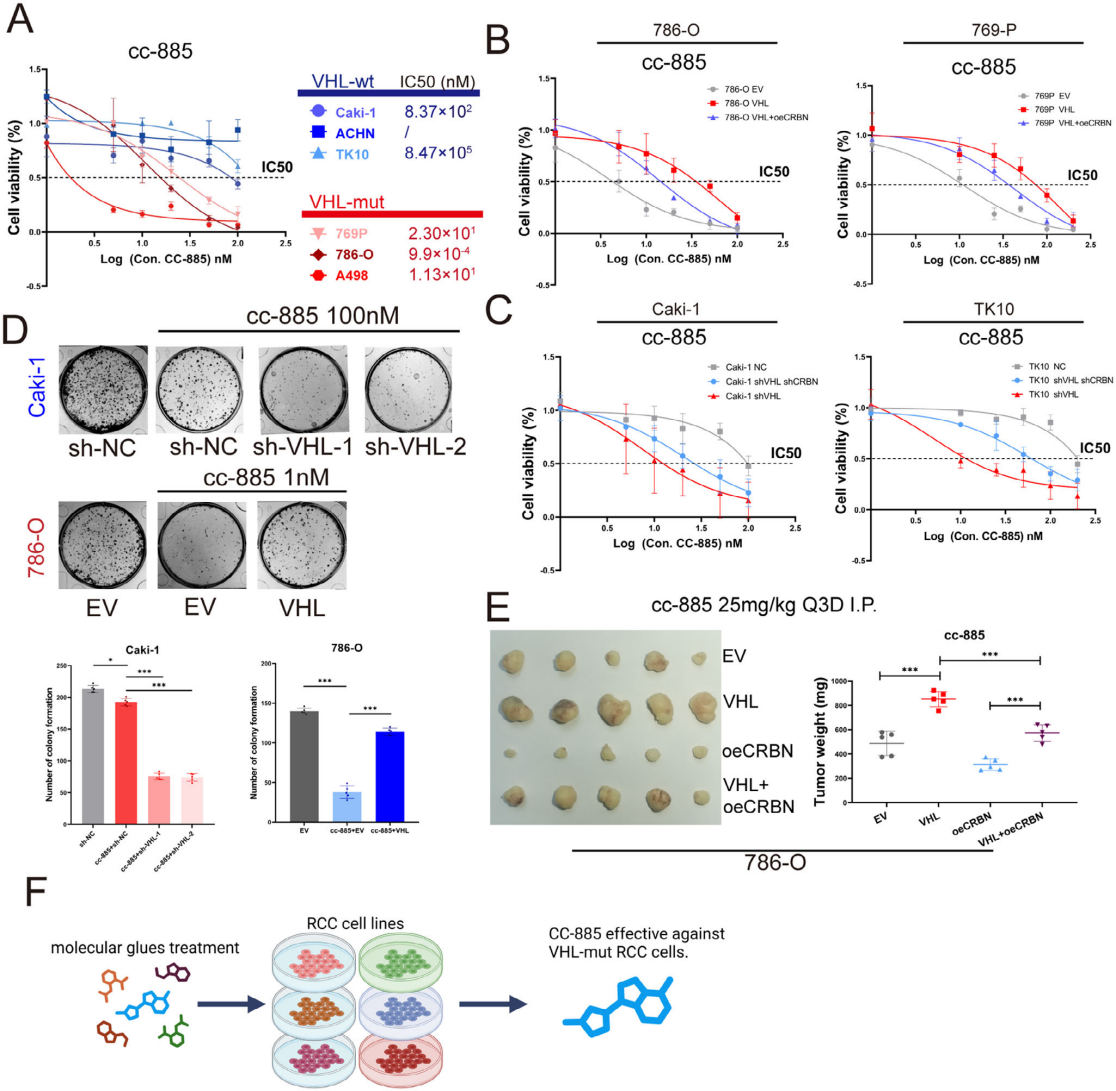
Selective Cytotoxicity of cc‐885 in VHL‐Deficient Renal Cancer Cells. (A) Dose‐response curves of cc‐885 in VHL‐wild‐type (wt) and VHL‐mutant (mut) RCC cell lines. Cell viability was measured after 72‐h treatment. Data are shown as mean ± SEM (n = 5). Statistical significance of differences between group means at each dose was determined by two‐way ANOVA with Šídák's multiple comparisons test. (B) Dose‐response curves of cc‐885 in 786‐O or 769‐P VHL‐mut ccRCC cells under genetic modulation: overexpression empty vector (EV), VHL overexpression (VHL), CRBN overexpression (oeCRBN). Data are shown as mean ± SEM (n = 5). Statistical significance of differences between group means at each dose was determined by two‐way ANOVA with Šídák's multiple comparisons test. (C) Dose‐response curves of cc‐885 in Caki‐1 or TK10 VHL‐wt ccRCC cells under genetic modulation: knockdown negative control (NC), VHL knockdown (shVHL), CRBN knockdown (shCRBN). Data are shown as mean ± SEM (n = 5). Statistical significance of differences between group means at each dose was determined by two‐way ANOVA with Šídák's multiple comparisons test. (D) Representative images (top) and corresponding quantification (bottom) of colony formation assays in Caki‐1 (VHL‐wild‐type) and 786‐O (VHL‐deficient) cells treated with DMSO or indicated concentrations of cc‐885. Caki‐1 cells were transduced with non‐targeting control (sh‐NC) or VHL‐targeting (sh‐VHL) shRNA. 786‐O cells stably expressing empty vector (EV) or VHL were used. Data are presented as mean ± SD from n = 5 independent experiments. Statistical comparisons among all groups within each cell line panel were performed using one‐way ANOVA followed by Tukey's multiple comparisons test. ^*^ *p* <0.05, ^***^ *p* <0.001. (E) Left: Representative xenograft tumors from 786‐O‐derived models. Right: Final tumor weights from each treatment group after cc‐885 treatment (25 mg/kg, Q3D, I.P.). Data are shown as mean ± SD (n = 5). Statistical significance was determined by ordinary one‐way ANOVA with Tukey's multiple comparisons test. ^***^ *p* <0.001. (F) Schematic model of cc‐885 selectivity in VHL‐deficient ccRCC.

To identify molecular glues specifically targeting VHL‐deficient ccRCC, we screened a panel of nine known molecular glues, including CRBN‐targeting thalidomide analogs and non‐thalidomide compounds, in VHL‐mutant (786‐O, 769‐P, A498) and VHL‐wildtype (Caki‐1, ACHN, TK10) renal cell carcinoma cell lines. Identification of the CRBN‐targeting thalidomide‐analog molecular glue cc‐885 as a selective compound for VHL‐deficient ccRCC was evidenced by significantly lower IC_50_ values in VHL‐mutant cell lines (Figure 1A; Figure S1A). Reconstitution of VHL in 786‐O or 769‐P cells led to a reduction in cc‐885 sensitivity, which was rescued by CRBN overexpression (Figure 1B). Conversely, VHL knockdown in wild‐type Caki‐1 or TK10 cells enhanced cc‐885 sensitivity, an effect that was reversed by CRBN knockout (Figure 1C).

In vitro and in vivo experiments further validated the selective antiproliferative effects of cc‐885 in VHL‐deficient cells. VHL reconstitution attenuated the antitumor efficacy of cc‐885, whereas CRBN overexpression restored drug sensitivity (Figure 1D,E; Figure S1B–D). These results indicate that VHL deficiency enhances CRBN‐dependent degradation pathways, conferring cc‐885's selective efficacy in VHL‐deficient ccRCC cells (Figure 1F).

### 2.2 ETS1 as a Novel CRBN‐Dependent Degradation Target of cc‐885

**FIGURE 2:**
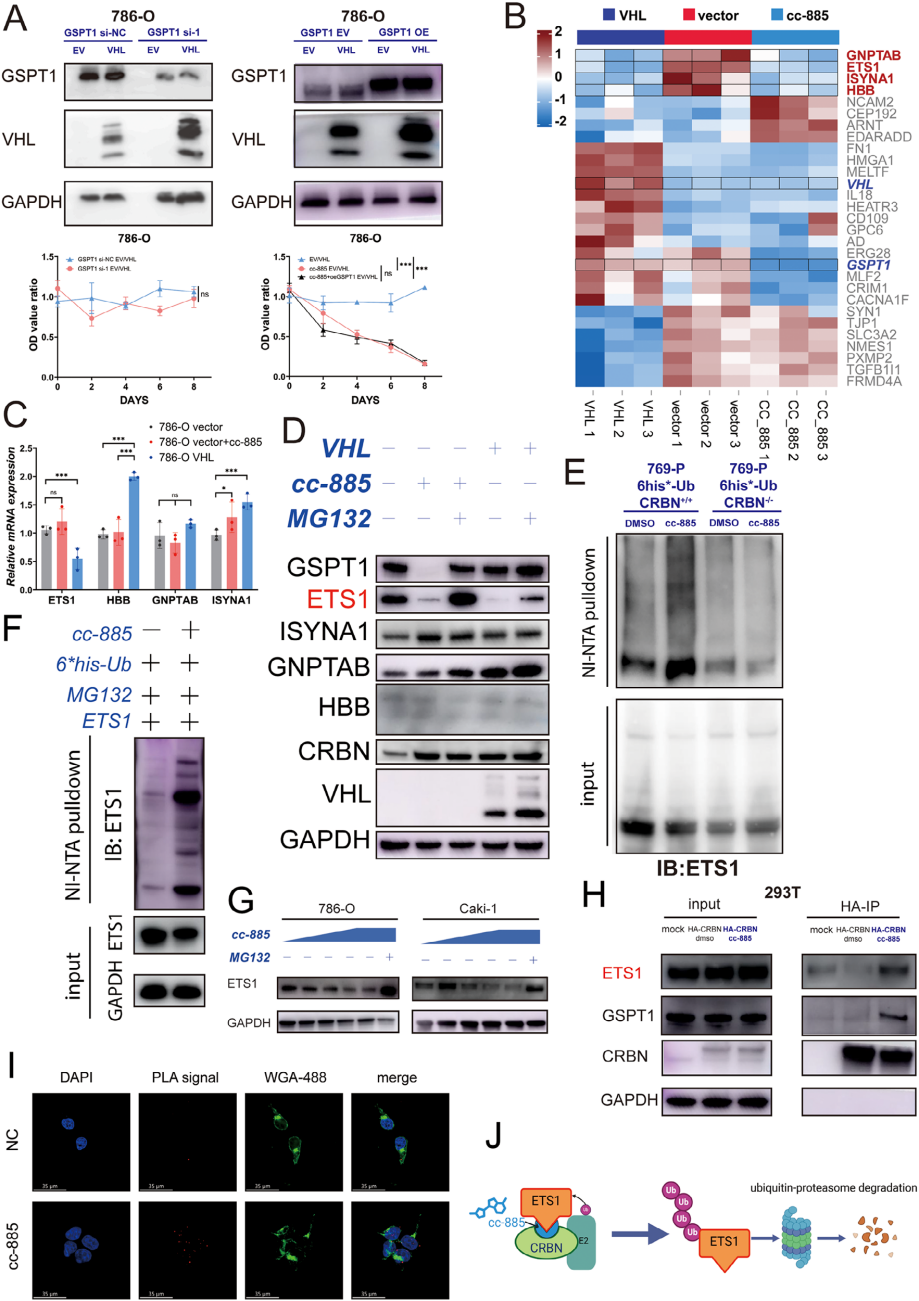
ETS1 is a CRBN‐dependent degradation target of cc‐885 in VHL‐deficient ccRCC cells. (A) (Left) 786‐O (VHL‐deficient) cells were transfected with non‐targeting control siRNA (si‐NC) or GSPT1‐targeting siRNA (si‐1), in combination with an empty vector (EV) or VHL‐expression plasmid. Western blots confirm GSPT1 knockdown and VHL restoration. Proliferation was monitored via CCK‐8 assay over 8 days. **(Right)** 786‐O cells stably overexpressing GSPT1 (GSPT1 OE) or EV were treated with DMSO or 10 nm cc‐885 in both VHL‐mutant and VHL‐reconstituted backgrounds. Western blots confirm GSPT1 overexpression and VHL status. The proliferation ratio of VHL‐mutant cells relative to VHL‐expressing cells did not differ significantly between cells with or without GSPT1 knockdown. Furthermore, the reduction in this VHL‐mutant/VHL‐expressing cell proliferation ratio induced by cc‐885 could not be rescued by GSPT1 overexpression. (data represent normalized OD ratios; mean ± SD, n = 3; ns, non‐significant, ^***^ *p* <0.001 one‐way ANOVA with Tukey's post‐hoc test). GAPDH was used as a loading control. (B) Heatmap of proteomic changes in 786‐O cells after VHL reconstitution or cc‐885 treatment (1 µm 8‐h). Expression levels are color‐coded from low (blue) to high (red). (C) RT‐qPCR analysis of candidate genes in 786‐O cells following VHL reconstitution or cc‐885 treatment (1 µm, 8 h). mRNA levels were normalized to GAPDH and expressed relative to the Vector + DMSO control. Mean ± SEM, n = 4; ns, non‐significant; ^*^ *p* <0.05, ^***^ *p* <0.001 (one‐way ANOVA with Dunnett's post‐hoc test). (D) Western blot analysis showing the expression levels of GSPT1, ETS1, ISYNA1, GNPTAB, HBB, CRBN, VHL in 786‐O cells treated with VHL reconstitution, 100 nm 24‐h cc‐885 or 20 µm 24‐h MG132. (E) Ni‐NTA pulldown assay of ubiquitinated ETS1 in 769‐P cells (wild‐type or CRBN knockout) transfected with 6xHis‐Ub and treated with MG132 and cc‐885 as indicated. Input samples are shown in the lower panel. (F) Ni‐NTA pulldown analysis of ubiquitinated exogenous ETS1 in 293T cells transfected with 6xHis‐Ub and treated with MG132 ± cc‐885. (G) Western blot analysis of ETS1 expression in 786‐O and Caki‐1 cells treated with increasing concentrations of cc‐885 or MG132. (H) Co‐immunoprecipitation (Co‐IP) in 293T cells transfected with HA‐CRBN and treated with DMSO or cc‐885. Cell lysates (Input) and HA‐precipitated fractions (HA‐IP) were analyzed by Western blot. (I) Representative Proximity Ligation Assay (PLA) images showing ETS1‐CRBN interactions (red foci) in 786‐O cells treated ± 10 nm cc‐885 for 12 h. Nuclei were stained with DAPI (blue) and membranes with WGA‐488 (green). (J) Schematic model of CRBN‐mediated ubiquitination and proteasomal degradation of ETS1 driven by cc‐885.

To determine the molecular targets of cc‐885 in VHL‐deficient cells, we assessed the known target, GSPT1 [13]. To determine whether the known CRBN‐dependent target GSPT1 mediates the selective effect of cc‐885, we first knocked down GSPT1 in 786‐O cells with or without VHL reconstitution. GSPT1 knockdown did not alter the relative proliferation ratio between VHL‐mutant and VHL‐reconstituted 786‐O cells (Figure 2A, left panel). Treatment with cc‐885 specifically reduced the viability of VHL‐mutant cells relative to their VHL‐reconstituted counterparts, leading to a decreased EV/VHL ratio. Importantly, this selective anti‐proliferative effect of cc‐885 was not rescued by forced overexpression of GSPT1 (Figure 2A, right panel). Together, these results indicate that GSPT1 is not the direct functional target responsible for the selective inhibition of VHL‐deficient ccRCC cells by cc‐885. TMT‐based proteomic analysis revealed that both cc‐885 treatment and VHL restoration downregulated several proteins, including GNPTAB, ETS1, ISYNA1, and HBB (Figure 2B; Figure S2B,C). Further analysis using quantitative PCR (qPCR) and western blotting showed that ETS1 mRNA and protein levels decreased specifically in VHL‐reconstituted cells, and this downregulation was rescued by the proteasome inhibitor MG132 (Figure 2C,D). These data suggest that ETS1 may be a downstream effector of cc‐885 sensitivity in VHL‐mutant cells.

CRISPR‐mediated CRBN knockout experiments confirmed that cc‐885's effects on ETS1 are CRBN‐dependent. Knockout of CRBN abolished cc‐885 sensitivity and ETS1 ubiquitination, as shown by ubiquitination assays and dose‐response experiments (Figure 2E–G; Figure S2D). Co‐immunoprecipitation (Co‐IP) and proximity ligation assays (PLA) demonstrated that cc‐885 treatment enhanced the interaction between CRBN and ETS1 (Figure 2H,I; Figure S2E). Collectively, these results established ETS1 as a functional target of cc‐885, which was degraded via CRBN‐mediated ubiquitination (Figure 2J).

### 2.3 ETS1 Y158 Mediates CRBN‐cc‐885 Ternary Complex Formation and Targeted Degradation

**FIGURE 3:**
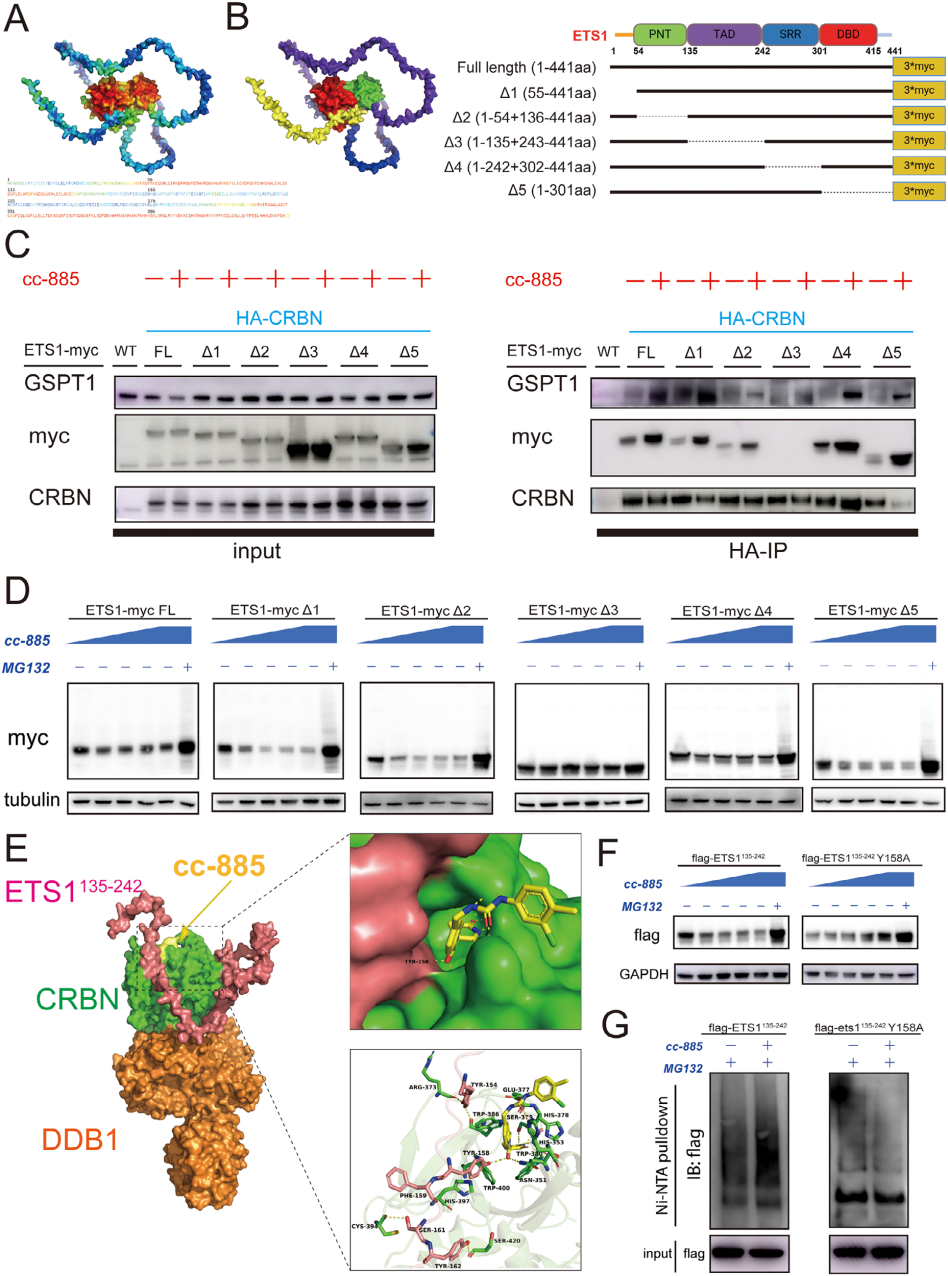
Structural and functional analysis of the CRBN‐ETS1 interaction mediated by cc‐885. (A) AlphaFold3‐predicted full‐length ETS1 structure (1‐441aa) colored by confidence score (b‐factor), with red indicating high confidence (stable domains) and blue low confidence (disordered regions). (B) Domain‐engineered ETS1 truncation mutants. Structural domains are color‐coded: Pointed Domain (PNT, green, 54–135aa), Transactivation Domain (purple, 135–242aa), Serine‐rich (SRR, blue, 242–301aa), and DNA‐binding domain (DBD, red, 301–415aa). Truncated mutants (Δ1‐Δ5) were C‐terminally tagged with 3×MYC. (C) In vitro HA‐CRBN immunoprecipitation (HA‐IP) assays with ETS1 truncation mutants. CRBN interaction requires the disordered region (135‐242aa; Δ3 mutant abolishes binding) and is enhanced by cc‐885 (1 µm). GSPT1 serves as a cc‐885‐CRBN binding positive control. (D) Gradient increased cc‐885‐induced degradation (24 h, 5–200 nm) of ETS1 truncation mutants analyzed by western blot. (E) Molecular docking model of ETS1 (135‐242aa) binding to CRBN‐DDB1‐cc‐885. Critical residue Y158 forms a hydrogen bond (yellow dashed line) with cc‐885. (F) Gradient increased cc‐885‐induced degradation (24 h, 5–200 nm) of ETS1^135‐242^ wt or Y158A mutants analyzed by western blot. (G) Ni‐NTA pulldown followed by FLAG immunoblot results the expression of ubiquitinated ETS1^135‐242^ wt or Y158A in 293T treated with MG132 and 6*his‐Ub w/wo cc‐885.

Next, we validated that Y158 is a critical residue essential for cc‐885‐mediated CRBN‐ETS1 binding and degradation through structural prediction, site‐directed mutagenesis, and molecular dynamics simulations. Using AlphaFold3 predictions, we identified stable Pointed (54‐135aa) and DNA‐binding (316‐441aa) domains in ETS1, while the 135–242aa region was predicted to be disordered (Figure 3A). We constructed a series of myc‐tagged ETS1 truncation mutants based on their protein domains (Figure 3B). Subsequent co‐immunoprecipitation (Co‐IP) experiments and dose‐dependent degradation assays with cc‐885 revealed that ETS1 truncation mutants lacking the 135–242aa domain (Δ3 mutant) were defective in both CRBN interaction and cc‐885‐mediated degradation (Figure 3C,D), demonstrating the essential role of this disordered region. Molecular docking analysis further identified a critical interaction within this domain, indicating a potential hydrogen bond between the side chain hydroxyl group of the ETS1‐Y158 residue and cc‐885 (Figure 3E). To functionally validate this structural prediction, we generated a point mutation that converted tyrosine 158 to alanine (Y158A). This single mutation completely abolished cc‐885‐induced ETS1 degradation and ubiquitination (Figure 3F,G), confirming the indispensable role of Y158 in the degradation mechanism.

**FIGURE 4:**
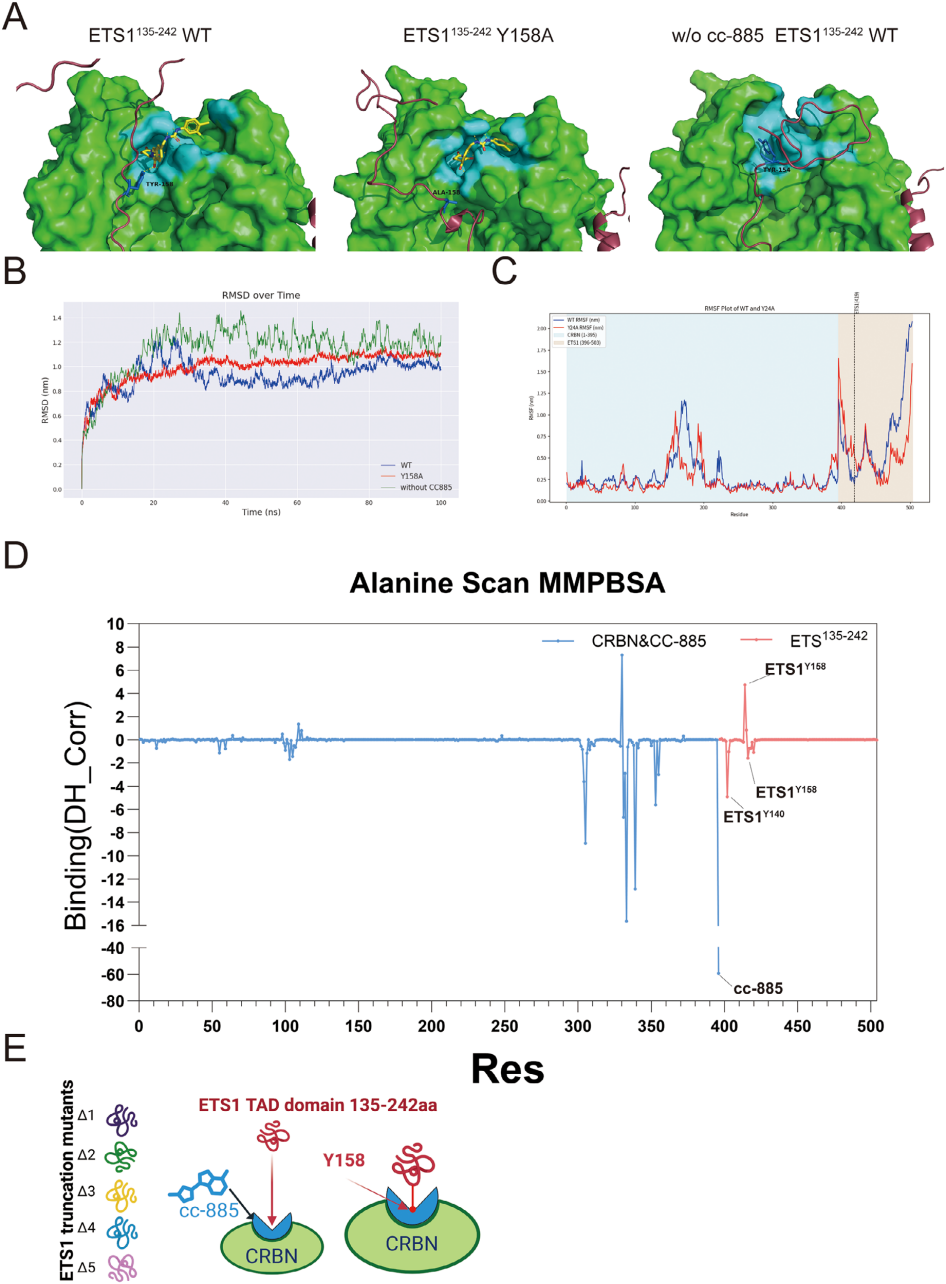
Molecular dynamics and binding energy analysis of the CRBN‐cc‐885‐ETS1 ternary complex. (A) Molecular models depict the ETS1^135‐242^ protein (dark red cartoon), wild‐type (WT), and Y158A mutant configurations, including states lacking cc‐885: Green color represents CRBN protein, therein blue areas indicate protein binding pocket regions; blue sticks represent either Y158 residue (WT), Y158A mutant residue, or binding pocket‐proximal residues; yellow dashed lines denote hydrogen bonds; and the yellow molecular entity corresponds to cc‐885 ligand. (B) Root Mean Square Deviation (RMSD) over time for ETS1^135‐242^ WT, Y158A, and without cc‐885. The *x*‐axis represents time in nanoseconds (ns), and the *y*‐axis shows RMSD values in nanometers (nm). The blue curve (WT), red curve (Y158A), and green curve (without CC885) depict the structural stability of each condition over time. (C) Residue‐specific Root Mean Square Fluctuation (RMSF) plot of ETS1^135‐242^ WT and Y158A mutant. The *x*‐axis indicates the position of amino acid residues, and the y‐axis displays RMSF values. The blue curve (WT) and red curve (Y158A) show the fluctuation of atoms in the two conditions, with significant differences at certain positions. (D) Alanine scan MMPBSA results for the binding free energy of ETS1^135‐242^ and CRBN with cc‐885. The *x*‐axis represents the residue number, and the y‐axis shows the binding free energy (kJ/mol). The blue curve (CRBN&cc‐885) and red curve (ETS^135‐242^) demonstrate the contribution of specific residues to binding energy, with notable negative peaks at cc‐885 or ETS1 Y140 or Y158 positions. (E) Summary of the effects of truncation mutants on the ternary complex stability. The TAD domain (135–242aa) and Y158 residue are essential for cc‐885‐mediated CRBN‐ETS1 binding.

Molecular dynamics simulations (100 ns) revealed the importance of the Y158 residue and cc‐885 molecule for the stability of the CRBN‐cc‐885‐ETS1 ternary complex. Simulations showed that in the stable wild‐type complex with cc‐885, the ETS1 Y158 residue interacted with both cc‐885. In contrast, the Y158A mutant complex and the wild‐type complex without cc‐885 exhibited structural distortions, where ETS1 either moved away from the binding pocket on CRBN or lacked key stabilizing interactions (Figure 4A). RMSD analysis confirmed this instability; the wild‐type complex with cc‐885 maintained the lowest and most stable RMSD values, whereas the Y158A mutant complex displayed a higher RMSD. Notably, the complex without cc‐885 exhibited significant RMSD fluctuations that failed to stabilize over the entire 100 ns simulation period (Figure 4B). RMSF analysis further showed that residues near the Y158 position in the Y158A mutant complex had increased flexibility, highlighting the specific role of Y158 in maintaining local and global complex stability (Figure 4C). MMPBSA analysis identified Y158 and Y140 as critical contributors to the binding energy, corroborating Y158's pivotal role in ternary complex stability (Figure 4D). Molecular dynamics simulations and experimental mutagenesis revealed that the ETS1 tyrosine residue Y158 and ligand cc‐885 are essential for the stability of the CRBN‐cc‐885‐ETS1 ternary complex and its degradation function, with Y158 forming a critical stabilizing interaction (Figure 4E).

### 2.4 cc‐885‐Mediated Selective Degradation of Active ETS1 Isoforms Reshapes Functional Balance

**FIGURE 5:**
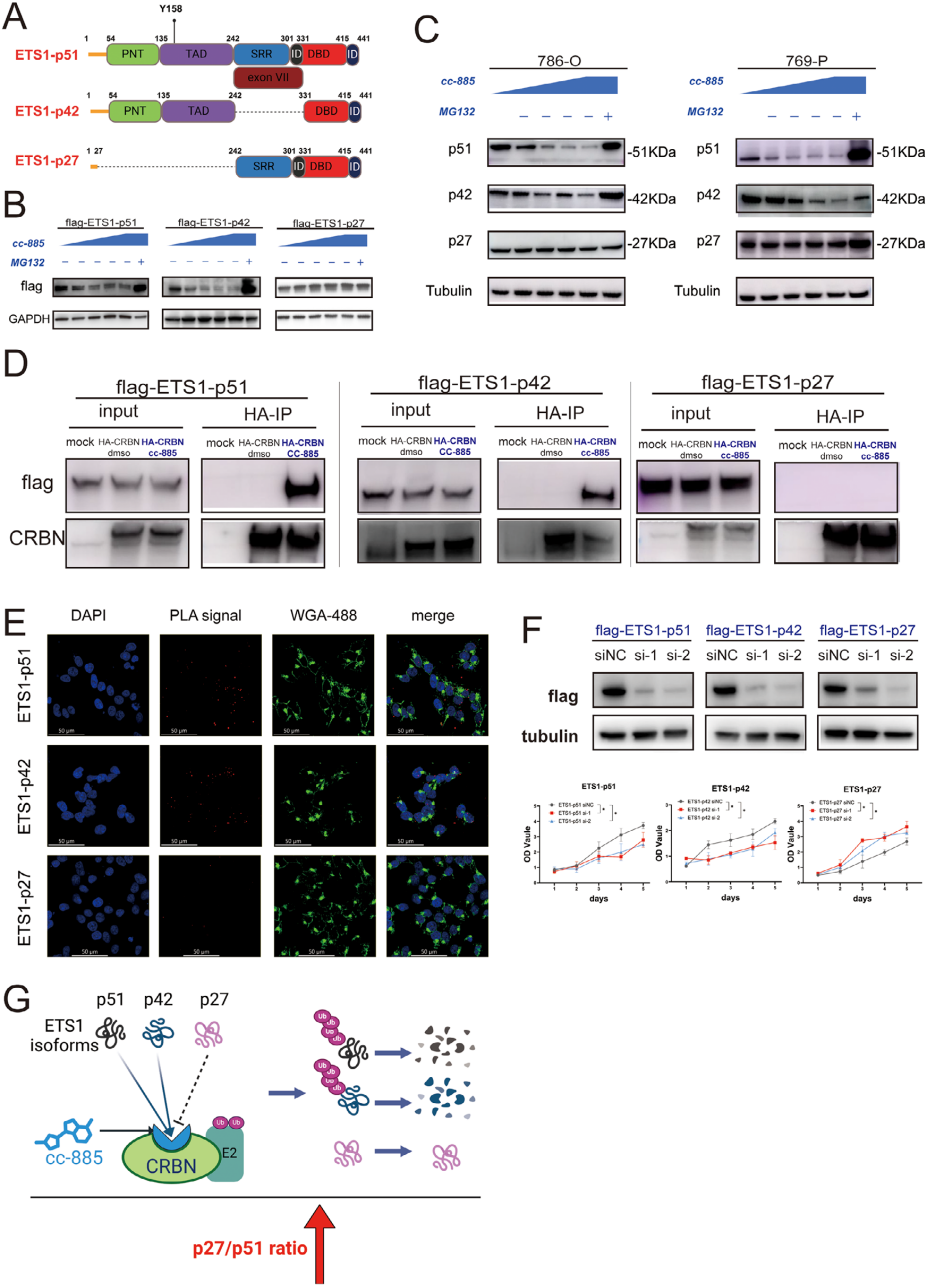
Isoform‐selective ETS1 degradation by cc‐885 alters functional balance in ccRCC cells. (A) Schematic representation of the structural domains of ETS1 isoforms (ETS1‐p51, ETS1‐p42, and ETS1‐p27). Each isoform is depicted with different colored rectangles representing their respective domains: PNT (green), TAD (purple), SRR (blue), ID (black), DBD (red), and exon VII (dark red box), with dashed lines indicating structurally absent regions in specific isoforms. PNT: Pointed Domain; TAD: Transactivation Domain; SRR: serine‐rich region: S251, S257, S282 and S285; IDs: Inhibitory Domains; DBD: DNA‐binding Domain. (B) Western blot analysis of ETS1 isoforms expression in 293T cells treated with gradient increased cc‐885 or MG132. (C) Western blot analysis of endogenous ETS1 isoform expression in 786‐O and 769‐P cells treated with increasing concentrations of cc‐885 or the proteasome inhibitor MG132. (D) HA‐CRBN immunoprecipitation (HA‐IP) in 293T cells co‐transfected with flag‐ETS1 isoforms treated w/wo cc‐885. (E) Representative images of DAPI staining (nuclei), proximity ligation assay (PLA, ETS1 isoforms‐CRBN interactions), WGA‐488 staining (membrane), and their merged views in 293T cells treated w/wo cc‐885 (5 nm, 12 h) (F) (Top) Western blot analysis of Flag‐ETS1‐p51, ‐p42, and ‐p27 expression in 786‐O cells transfected with non‐targeting (si‐NC) or two independent ETS1‐variants targeting siRNAs (si‐1, si‐2). Blots are representative of n = 3 independent experiments. (Bottom) Corresponding cell proliferation curves measured by CCK‐8 assay. Data points represent the mean ± SEM of n = 5 biological replicates. Statistical significance was determined by two‐way ANOVA with Šídák's multiple comparisons test comparing each siRNA group to the si‐NC control over time; ^*^ *p* <0.001. (G) Schematic model of cc‐885‐mediated isoform‐selective degradation. cc‐885 degrades transcriptionally active p51/p42 via CRBN, shifting the balance toward dominant‐negative p27 (red arrows), thereby suppressing oncogenic signaling.

The transcription factor ETS1 primarily exists as three major splice variants: the full‐length p51 isoform, p42, which lacks exon VII, and p27, which is deficient in the pointed domain and transactivation domain (TAD) (Figure 5A). Functionally, p51 contains autoinhibitory domains (N‐ID and C‐ID) flanking its DNA‐binding domain (DBD), and its transcriptional activity is modulated by phosphorylation within its Serine‐Rich Region (SRR). p42, lacking the SRR and part of the N‐ID, typically acts as a constitutively active transcriptional activator, and p27, devoid of the TAD, functions as a dominant‐negative isoform by competitively inhibiting DNA binding and transactivation by p51 and p42 [14, 15]. Critically, the p27/p51 ratio is a key determinant of cellular fate [16]. Sequence analysis of these isoforms revealed that the CRBN‐binding molecule cc‐885 selectively degrades ETS1 p51 and p42, which contain the 135–242 amino acid domain, but not the region‐lacking p27 (Figure 5B). Consistently, cc‐885 treatment selectively reduced the endogenous ETS1 isoforms protein levels of p51 and p42, but not p27, in VHL‐deficient ccRCC cells (786‐O and 769‐P). (Figure 5C). This selectivity was confirmed by co‐immunoprecipitation (Co‐IP) and Proximity Ligation Assay (PLA) experiments, which demonstrated that cc‐885 induces a direct interaction between CRBN and p51/p42, but not p27 (Figure 5D,E). Functional studies aligned with these findings: knockdown of the activating isoforms p51 and p42 inhibited cell proliferation, whereas overexpression of dominant‐negative p27 promoted tumor growth (Figure 5F). Collectively, these results demonstrate that cc‐885 reshapes the functional balance of ETS1 isoforms by selectively degrading the transcriptionally active p51 and p42. This shift in isoform equilibrium, increasing the relative abundance of inhibitory p27 and decreasing active isoforms, impeded the transcriptional activation of ETS1‐dependent pro‐survival genes, ultimately suppressing cell proliferation (Figure 5G).

### 2.5 ETS1‐EPAS1 Interaction Drives Oncogenic Transcription

**FIGURE 6:**
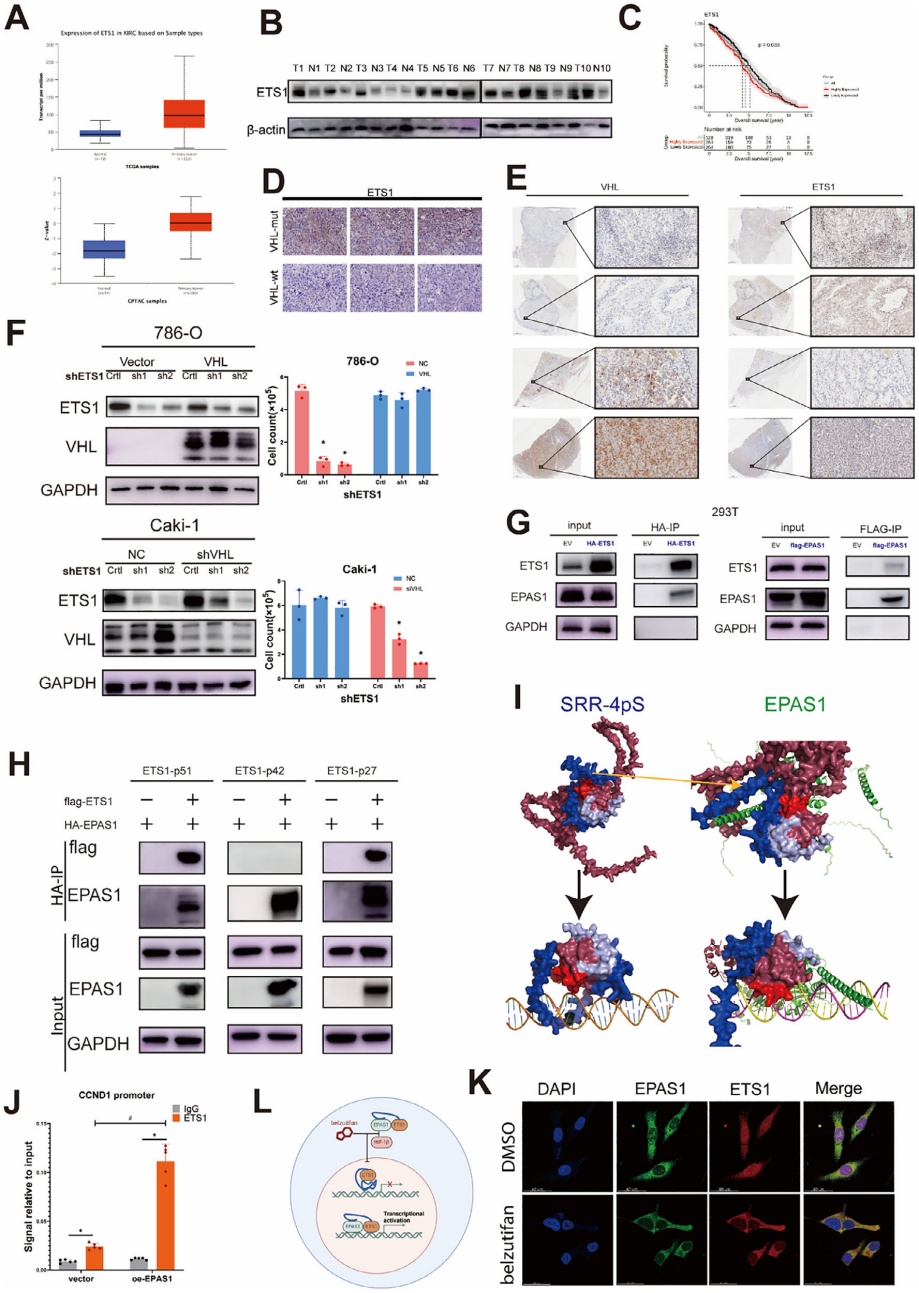
ETS1‐EPAS1 interaction drives oncogenic transcription in ccRCC. (A) ETS1 mRNA expression in TCGA and protein expression in CPTAC of KIRC. (B) Western blot analysis of ETS1 isoform expression in paired renal cell carcinoma (RCC) and adjacent normal tissues (n = 10 samples). (C) Kaplan–Meier survival curve comparing the survival probability of patients with high ETS1 expression versus low ETS1 expression. Patients with high ETS1 expression show significantly lower survival rates. (D) Immunohistochemical staining of ETS1 in 786‐O (vector vs. VHL‐ reconstitution) xenograft tumors. (E) Representative immunohistochemical (IHC) staining images of VHL and ETS1 in serial sections from 10 paired human clear cell renal cell carcinoma (ccRCC) samples. The left panel shows VHL staining, and the right panel shows corresponding ETS1 staining in adjacent sections from the same tumor region. VHL‐deficient tumors exhibit low or absent VHL staining but high nuclear ETS1 expression, whereas VHL‐proficient tumors show strong VHL signal and weak or absent ETS1 staining. Scale bars, 25 or 100 µm. (F) (Left) Western blot analysis confirming ETS1 and VHL reconstitution or knockdown efficiency in 786‐O (VHL‐mutant) and Caki‐1 (VHL‐wild‐type) cell lines. Blots are representative of n = 3 independent experiments. (Right) Corresponding cell count at 96 h post‐transfection. Data are presented as mean ± SEM (n = 5). Statistical significance was determined by two‐way ANOVA with Šídák's multiple comparisons test comparing each sh‐ETS1 group to the respective shNC control within the same cell line; ^*^ *p* <0.001. (G) Co‐immunoprecipitation (Co‐IP) experiment in 293T cells using HA‐tagged ETS1 and FLAG‐tagged EPAS1. (H) HA‐EPAS1 immunoprecipitation (HA‐IP) in 293T cells co‐transfected with flag‐ETS1 isoforms. (I) Molecular mechanism of phospho‐regulated ETS1‐EPAS1‐DNA complex assembly: (Left) Phosphorylation of SRR domain serine residues (S251/S257/S282/S285) triggers cooperative autoinhibition, where the phospho‐SRR (blue) and N‐terminal inhibitory domain (blue) sterically occlude the DNA‐binding domain (DBD, red). (Right) EPAS1 binding induces allosteric rearrangement of phospho‐ETS1, releasing autoinhibitory constraints. This exposes DBD for cooperative DNA binding with EPAS1 at adjacent GGAA/T and hypoxia‐response elements. (J) EPAS1 potentiates ETS1 binding to the CCND1 promoter. Chromatin immunoprecipitation (ChIP) assays were performed in 786‐O cells transfected with empty vector (EV) or EPAS1 overexpression plasmid (oe‐EPAS1), using an anti‐ETS1 antibody or normal IgG (negative control). Enrichment of the CCND1 promoter region was quantified by qPCR and is presented as percentage of input. Data are presented as mean ± SEM of n = 4 independent ChIP experiments. Statistical significance was determined by unpaired, two‐tailed Student's t‐test comparing the oe‐EPAS1 group to the EV control group; ^*^ *p* <0.001, ^#^ *p* <0.001. (K) Merge images of DAPI, EPAS1, and ETS1 immunofluorescence staining. The images show the nuclear localization (DAPI staining) and the subcellular localization of EPAS1 and ETS1 under DMSO and belzutifan treatment. Belzutifan treatment results in changes in the localization of EPAS1 and ETS1. (L) Schematic diagram summarizing the mechanism of EPAS1 and ETS1 in transcriptional activation.

Comprehensive analysis of clinical data from The Cancer Genome Atlas (TCGA) and our institutional cohort demonstrated that elevated ETS1 expression is prevalent in clear cell renal cell carcinoma (ccRCC) and is significantly correlated with poor patient prognosis (Figure 6A–C; Figure S3A). Immunohistochemical (IHC) evaluation of xenograft models revealed that restoration of VHL function suppressed ETS1 expression (Figure 6D). In line with this, analysis of primary human ccRCC tissues confirmed an inverse correlation, showing that tumors with low VHL expression exhibited high ETS1 levels (Figure 6E). Furthermore, hypoxia enhanced the transcriptional activation of ETS1 in VHL‐reconstituted cells (Figure S3B), supporting a mechanistic link between VHL loss/HIF stabilization and ETS1 upregulation. GO enrichment analysis indicated ETS1 in cellular “ responses to oxygen levels” (Figure S3C), and DepMap analysis confirmed ETS1 dependency in ccRCC, revealing a strong correlation with EPAS1 expression (Figure S3E–G). Mechanistically, EPAS1 overexpression increased both ETS1 transcript and protein levels, whereas the EPAS1 nuclear translocation inhibitor belzutifan abrogated this upregulation (Figure S3D), indicating the EPAS1‐mediated transcriptional control of ETS1. Functionally, ETS1 knockdown specifically suppressed cell viability in VHL‐deficient or VHL‐knockdown cells but not in VHL‐wild‐type cells (Figure 6F). By establishing the roles of EPAS1 and ETS1 as transcriptional co‐factors in ccRCC [17], Co‐IP confirmed their direct physical interaction (Figure 6G), with isoform‐specific analysis showing that EPAS1 binds to p51 and p27, but not p42 isoforms (Figure 6H). Consistent with the well‐established ETS1 regulation model reported in previous studies [18, 19], our structural analysis indicated that EPAS1 binding relieves ETS1 autoinhibition, thereby exposing its DNA‐binding domain and enhancing transcriptional activity (Figure 6I). Chromatin immunoprecipitation (ChIP) assays revealed that EPAS1 expression potentiated ETS1 binding to the CCND1 promoter (Figure 6J). Critically, belzutifan disrupted both EPAS1 nuclear translocation and ETS1 nuclear localization (Figure 6K), establishing a spatially coordinated role in driving ccRCC oncogenesis. (Figure 6L).

### 2.6 Combined Anti‐Tumor Effects of ETS1 Degradation and EPAS1 Inhibition

**FIGURE 7:**
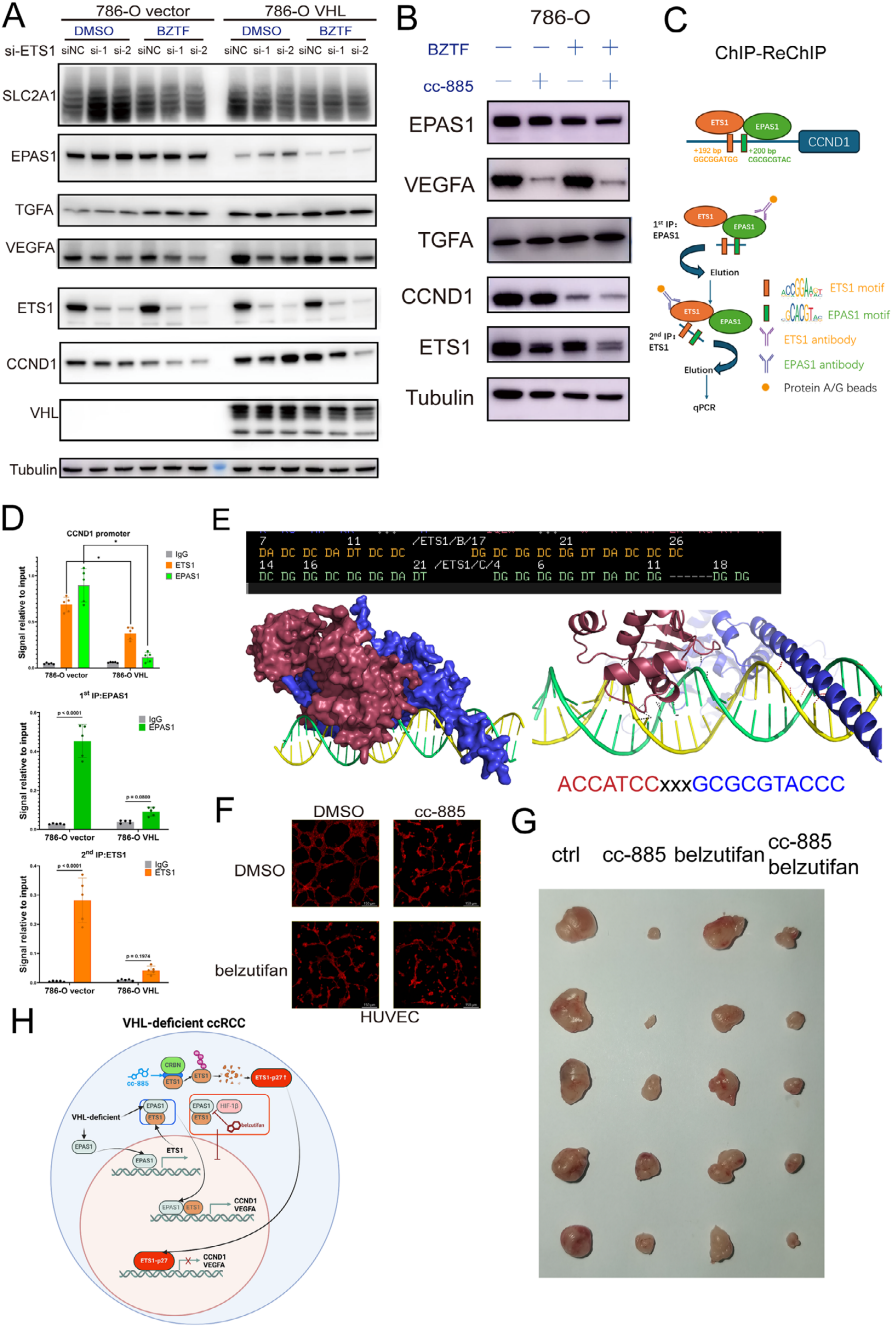
Dual inhibition of oncogenic signaling by cc‐885 and belzutifan in VHL‐deficient ccRCC. (A) Western blot analysis of protein expressions in 786‐O cells treated with DMSO or belzutifan (BZTF), and transfected with ETS1 siRNA. (B) western blot analysis of protein expression in 786‐O cells treated with BZTF and/or cc‐885. (C) Schematic representation of the ChIP‐ReChIP assay. The first Chromatin immunoprecipitation was performed using an EPAS1 antibody, followed by elution and a second IP using an ETS1 antibody. The final eluates were analyzed by qPCR. (D) Sequential chromatin immunoprecipitation (ChIP‐ReChIP) analysis was performed in 786‐O cells (expressing empty vector or reconstituted with VHL). The final DNA enrichment at the CCND1 promoter region is presented as percentage of input. Data are shown as mean ± SEM from n = 4 independent experiments. Statistical significance was determined by two‐way ANOVA with Šídák's multiple comparisons test; ^*^ *p* <0.05, comparing the ChIP‐ReChIP (EPAS1→ETS1) signal to the IgG control within each cell line. (E) Structural model of ETS1 DNA‐binding domain (red) and EPAS1 (blue) interacting with the CCND1 promoter. (F) Fluorescence microscopy analysis of tube formation in HUVECs co‐cultured with conditioned media from 786‐O cells treated with DMSO, cc‐885, or belzutifan, stained with rhodamine‐phalloidin. (G) In vivo growth of 786‐O xenograft tumors following intraperitoneal administration of DMSO, cc‐885, and/or belzutifan. (H) Schematic model of the combination of cc‐885 and belzutifan suppressing angiogenesis via ETS1‐p27‐mediated inhibition of VEGFA/CCND1.

Building on our characterization of the ETS1‐EPAS1 oncogenic axis, we further investigated the therapeutic efficacy of combining the CRBN‐targeting molecule cc‐885 with the EPAS1 inhibitor belzutifan in VHL‐deficient ccRCC models. In vitro studies demonstrated that combined ETS1 knockdown and belzutifan treatment combinedly suppressed the expression of key downstream oncogenic targets, VEGFA and CCND1. Notably, this enhanced effect was significantly attenuated by VHL reconstitution (Figure 7A). Strikingly, co‐treatment with cc‐885 and belzutifan achieved maximal suppression of both VEGFA and CCND1, as well as ETS1 itself, indicating dual blockade of the pathway at both the transcriptional regulator (ETS1) and effector (target gene) levels. To mechanistically define the cooperative action of EPAS1 and ETS1 at the target promoters, we performed sequential Chromatin Immunoprecipitation (ChIP‐ReChIP), which confirmed the direct co‐occupancy of EPAS1 and ETS1 in the CCND1 promoter region (Figure 7D). Complementary molecular docking analysis identified a novel composite DNA‐binding motif accommodating both the EPAS1 and ETS1 consensus sequences, providing a structural rationale for their functional cooperation (Figure 7E). Functionally, this combination strategy translated to significant phenotypic effects: in vitro, cc‐885 plus belzutifan potently inhibited endothelial tube formation, reflecting impaired angiogenesis driven by VEGFA suppression; in vivo, the dual treatment regimen markedly reduced tumor growth in VHL‐deficient ccRCC xenograft models compared to monotherapy or vehicle control (Figure 7F,G). Collectively, these findings highlight the therapeutic potential of a dual‐targeting strategy utilizing cc‐885 to degrade transcriptionally active ETS1 isoforms along with belzutifan to inhibit EPAS1 function, effectively disrupting the core oncogenic partnership in VHL‐deficient ccRCC.

## 3 Discussion

This study establishes cc‐885, a molecular glue degrader, as a precise therapeutic strategy for VHL‐deficient clear cell renal cell carcinoma (ccRCC) by selectively targeting the transcription factor ETS1 for degradation. Mechanistically, cc‐885 exploits a non‐canonical degron within ETS1's disordered 135–242aa domain, where the tyrosine residue Y158 forms a critical hydrogen bond with cc‐885 to stabilize the CRBN‐cc‐885‐ETS1 ternary complex, a requirement abolished by the Y158A mutation. Notably, we observed a dose‐dependent accumulation of the ETS1‐Y158A mutant upon cc‐885 treatment, which can be mechanistically explained by the combination of degradation escape and cellular homeostatic feedback. The Y158A mutation disrupts the critical hydrogen bond with cc‐885, abrogating the recruitment of the mutant to the CRBN E3 ligase complex and rendering it completely refractory to cc‐885‐induced proteasomal degradation. As cc‐885 treatment depletes the endogenous wild‐type ETS1, cells activate a homeostatic feedback response to upregulate ETS1 transcription to compensate for the loss of functional ETS1 activity. Since the Y158A mutant evades degradation, this compensatory upregulation leads to a pronounced accumulation of the stable, functionally competent mutant protein. This observation also highlights a potential clinical implication: ETS1 mutations at the Y158 residue could confer resistance to cc‐885 therapy, as such mutants would escape targeted degradation and maintain oncogenic transcriptional activity. cc‐885 exhibits exquisite isoform selectivity, degrading transcriptionally active p51/p42 isoforms while sparing dominant‐negative p27, thereby shifting the p27/p51 balance to suppress pro‐survival gene networks [16].

Of note, as a previously reported molecular glue degrader, cc‐885 has been documented to target other substrates such as GSPT1, which raises potential concerns regarding off‐target effects. In this study, our GSPT1 knockdown and overexpression rescue experiments confirmed that GSPT1 does not mediate the selective anti‐tumor effect of cc‐885 in the context of VHL‐deficient ccRCC, and ETS1 is the core functional target responsible for the therapeutic efficacy. Nevertheless, other potential off‐target substrates of cc‐885 may still have implications for clinical translation, and future studies are warranted to further evaluate its off‐target effects and potential toxicity in normal tissues, to define the safety window for its clinical application.

In addition to direct degradation, we uncovered an essential oncogenic partnership between ETS1 and EPAS1. EPAS1 transcriptionally upregulates ETS1 while physically interacting with exon VII to relieve its autoinhibition, forming a feed‐forward loop that drives oncogenes such as CCND1 and VEGFA. This interdependence explains the VHL context‐specificity of ETS1 dependency and positions the ETS1‐EPAS1 complex as a central hub for hypoxia signaling in ccRCC. Importantly, prior studies corroborate the functional conservation of this partnership; ETS1‐EPAS1 cooperativity was shown to co‐activate endothelial‐specific genes (e.g., Flk‐1 and VE‐cadherin) and promote tyrosine kinase inhibitor (TKI) resistance by amplifying pro‐survival transcriptomes [17, 20, 21].

Notably, the autoinhibition model of ETS1 SRR phosphorylation proposed in this study is built on the well‐validated mechanism reported in previous work [18, 19], which has systematically characterized that the SRR of ETS1 functions as an intrinsic inhibitory module: the disordered SRR region can transiently associate with the ETS DNA‐binding domain to block DNA binding, and this autoinhibitory effect is further enhanced by phosphorylation of the adjacent serines.

Although this study has not provided direct experimental evidence for the endogenous phosphorylation status of ETS1 SRR in our experimental system, the existing evidence from our study, combined with the well‐established conserved mechanism from previous reports, strongly supports that EPAS1 can relieve this autoinhibition to enhance the transcriptional activity of ETS1 [17]. Future studies will be needed to directly validate the phosphorylation event in this specific context to further confirm this mechanism.

By leveraging this axis, combining cc‐885 with the EPAS1 inhibitor belzutifan achieved a potent combined effect. This dual‐targeting strategy implements a vertical pathway blockade: cc‐885 degrades the transcriptional activator ETS1, whereas belzutifan inhibits EPAS1 nuclear translocation and co‐localization [22, 23]. Co‐treatment maximally suppressed downstream oncogenic targets, impaired angiogenesis, and significantly reduced tumor growth in VHL‐deficient models beyond monotherapy effects. （Figure 7H）

Regarding the clinical translation of this combination therapy, our study indicates that VHL mutation status can serve as a core predictive biomarker for cc‐885 treatment sensitivity, which provides a theoretical basis for subsequent patient stratification. However, there are still several unresolved issues for the clinical application of this combination strategy: First, CRBN‐dependent resistance mechanisms, such as loss‐of‐function mutations or downregulated expression of CRBN, may limit the long‐term efficacy of this molecular glue degrader. Second, the tissue tolerance of this combination regimen needs further evaluation to clarify whether normal tissues will be affected by the toxicity of combined medication, and future preclinical studies need to conduct an in‐depth exploration of these issues.

The structural blueprint of the cc‐885 mechanism opens avenues for targeting disordered domains in other transcription factors. Clinically, the cc‐885/belzutifan combination warrants an expedited evaluation as a transformative strategy for aggressive ccRCC.

## 4 Methods

### 4.1 Cell Lines and Culture Conditions

Human clear cell renal cell carcinoma (ccRCC) cell lines including 786‐O (ATCC CRL‐1932; RRID: CVCL_1051), 769‐P (ATCC CRL‐1933; RRID: CVCL_1050), A498 (ATCC HTB‐44; RRID: CVCL_1056), OSRC2 (Cell Bank of the Chinese Academy of Sciences; RRID: CVCL_1626), RCCJF (Cell Bank of the Chinese Academy of Sciences; RRID: CVCL_2860), Caki‐1 (ATCC HTB‐46; RRID: CVCL_0234), Caki‐2 (ATCC HTB‐47; RRID: CVCL_0235), ACHN (ATCC CRL‐1611; RRID: CVCL_1067), TK10 (Cell Bank of the Chinese Academy of Sciences; RRID: CVCL_1733), along with human embryonic kidney 293T cells (ATCC CRL‐3216; RRID: CVCL_0063) were cultured in DMEM/F12 or RPMI‐1640 medium supplemented with 10% fetal bovine serum (FBS), 100 U/mL penicillin, and 100 µg/mL streptomycin at 37 °C in 5% CO_2_; all cell lines were regularly tested using the MycoAlert Mycoplasma Detection Kit (Lonza, Cat# LT07‐318) and confirmed free of mycoplasma contamination throughout the study.

### 4.2 Cell Viability (CCK8 Assay) and Colony Formation Assay

Cell proliferation was assessed using the Cell Counting Kit‐8 (CCK8) assay. Cells were seeded in 96‐well plates at a density of 5000 cells/well and treated with cc‐885 at various concentrations (0–10 µm) for 72 h. After treatment, 10 µL of CCK8 reagent was added to each well, and the absorbance was measured at 450 nm using a microplate reader. Cell viability was calculated as a percentage of that of the untreated control.

For the colony formation assay, cells were seeded in six‐well plates at an initial density of 500 cells per well. After cell attachment, cells were treated with the indicated concentrations of cc‐885 and cultured for 14 days. At the end of the culture period, cells were fixed with 4% paraformaldehyde for 15 min, followed by staining with 0.1% crystal violet for 10 min. The number and area of the colonies were quantified using ImageJ software, and statistical analysis was performed using an unpaired two‐tailed t‐test to compare the differences between groups. All experiments were independently repeated three times to ensure reproducibility.

### 4.3 Western Blot Analysis

Protein expression was analyzed by western blotting. Cells were lysed in radioimmunoprecipitation assay (RIPA) buffer containing protease and phosphatase inhibitors. Protein concentrations were determined using the bicinchoninic acid assay. Equal amounts of protein were separated by SDS‐PAGE and transferred to PVDF membranes. Membranes were then incubated with primary antibodies. After incubation with HRP‐conjugated secondary antibodies, protein bands were visualized using ECL reagent and quantified by densitometry. The use of human renal tumor specimens for Western blot analysis was approved by the Ethics Committee of Sun Yat‐sen University, and informed consent was obtained from all patients.

### 4.4 Immunohistochemistry (IHC)

Tumor xenograft tissues were fixed in 4% paraformaldehyde, paraffin‐embedded, and sectioned (5 µm). Immunohistochemistry (IHC) staining was performed using antibodies against ETS1. The sections were incubated with primary antibodies overnight at 4 °C, followed by incubation with biotinylated secondary antibodies and an avidin‐biotin complex. Staining was visualized with DAB substrate, and images were captured using a light microscope. Quantification of positive cells was performed using the ImageJ software. The use of human renal tumor specimens for IHC analysis was approved by the Ethics Committee of Sun Yat‐sen University, and informed consent was obtained from all patients.

### 4.5 CRISPR/Cas9 Knockout

CRBN‐knockout cell lines were generated using CRISPR/Cas9 technology. sgRNAs targeting specific exons of CRBN were designed using online tools and cloned into the lentiCRISPRv2 plasmids. Cells were transfected with the plasmids using Lipofectamine 3000, and stable clones were selected using puromycin (2 µg/mL). Knockout efficiency was verified using Sanger sequencing and western blot analysis.

### 4.6 RNA/DNA Isolation, Followed by RT‐qPCR Analysis and Sanger Sequencing

Total RNA was extracted from the cells using TRIzol reagent, and genomic DNA was isolated using a standard phenol‐chloroform method. RNA was reverse‐transcribed into cDNA using a SuperScript IV reverse transcriptase kit. Quantitative real‐time PCR (qPCR) was performed using SYBR Green Master Mix to quantify the mRNA levels of target genes. The primer sequences were designed using Primer‐BLAST. Sanger sequencing was used to confirm CRISPR‐mediated mutations and validate the knockout clones.

### 4.7 Chromatin Immunoprecipitation (ChIP) and Re‐ChIP

ChIP assays were performed to analyze ETS1 binding to the target gene promoters. Cells were cross‐linked with formaldehyde, lysed, and chromatin was sonicated to generate 200–500 bp fragments. Immunoprecipitation was performed using anti‐ETS1 antibodies, followed by overnight incubation with protein A/G beads. DNA was purified and subjected to qPCR using primers targeting ETS1‐binding sites in the VEGFA and CCND1 promoters. Re‐ChIP was performed to confirm the sequential binding of ETS1 and EPAS1 using sequential immunoprecipitating with antibodies against each protein.

### 4.8 Reagents and Transfection

Plasmids expressing FLAG‐tagged ETS1 isoforms (p27, p51, and p42) or mutants (e.g., Y158A) were transfected into cells using Lipofectamine 2000 according to the manufacturer's protocol. Transfection efficiency was assessed using western blotting or fluorescence microscopy. cc‐885 was dissolved in DMSO and diluted in the culture medium to achieve the desired concentrations. Control cells were treated with an equal volume of DMSO.

### 4.9 Proteomics (Tandem Mass Tag, TMT)

Changes in global protein expression were analyzed using TMT labeling. Cells treated with cc‐885 or the control were lysed, proteins were digested with trypsin, and labeled with TMT reagents. The peptides were fractionated by high‐pH reverse‐phase chromatography and analyzed using LC‐MS/MS. Data were processed using MaxQuant software to identify differentially expressed proteins.

### 4.10 Structural Biology and Binding Studies

The ternary complex structure of CRBN‐cc‐885‐ETS1 was initially predicted using AlphaFold3 to identify ETS1 domains, revealing a stable pointed domain (54–135 aa), DNA‐binding domain (316–441 aa), and a disordered region (135–242 aa). Molecular docking studies were performed with HADDOCK to model the interactions between cc‐885 and the CRBN‐ETS1 interface, identifying a critical hydrogen bond between the hydroxyl groups of ETS1‐Y158 and cc‐885. To validate the domain functionality, a series of myc‐tagged ETS1 truncation mutants (full‐length, Δ1–Δ5) were engineered based on domain boundaries and tested via co‐immunoprecipitation (Co‐IP) and dose‐dependent degradation assays. Site‐directed mutagenesis was used to generate the ETS1‐Y158A point mutant for mechanistic validation. Molecular dynamics (MD) simulations (100 ns duration, AMBER20 force field) were used to compare ternary complex stability across four systems: (1) wild‐type CRBN‐cc‐885‐ETS1, (2) Y158A mutant complex, (3) wild‐type without cc‐885, and (4) Y158A without cc‐885. The trajectories were analyzed for root‐mean‐square deviation (RMSD), root‐mean‐square fluctuation (RMSF), and Molecular Mechanics/Poisson‐Boltzmann Surface Area (MMPBSA) binding energy calculations. Simulations were visualized using PyMOL, with key interactions quantified using the gmx mmbsa module in GROMACS.

### 4.11 Cellular Immunoprecipitation and In Vivo Ubiquitination Assay

Cells were lysed in Buffer B (50 mM Tris (pH 7.4), 150 mM NaCl, 0.5% NP‐40, one tablet of Complete ULTRA protease inhibitor cocktail (Roche), and one tablet of PhosSTOP phosphatase inhibitor cocktail (Roche)). Whole‐cell lysates were clarified by centrifugation at top speed for 10 min. To identify proteins interacting with CRBN/cc‐885, cell lysates were incubated with 10 µM cc‐885 or DMSO for 2h. HA‐CRBN complexes were then captured using 30µL HA Magnetic Beads (MCE, Cat. No. HY‐K0201A) overnight. Beads were washed six times with Buffer B containing cc‐885 or DMSO, and bound proteins were eluted by boiling in 2× LDS loading buffer for subsequent SDS‐PAGE separation and immunoblot or mass spectrometry analysis.

Cells were cultured in 10‐cm dishes and transfected with 12 µg of pcDNA3‐6×His‐Ub plasmid. At 48 h post‐transfection, cells were treated with 10 µM cc‐885 and 10 µM MG132 for 8 h. Cells were harvested, washed with ice‐cold PBS, and lysed in denaturing Buffer A (6 M guanidine–HCl, 0.1 M Na_2_HPO_4_/NaH_2_PO_4_, 20 mM imidazole, pH 8.0). Whole‐cell extracts were sonicated and incubated with 30 µL of GenScript MagBeads Ni‐NTA (Cat. No. L00295) at room temperature for 4 h. Beads were sequentially washed with Buffer A, Buffer A/TI (1:3 ratio of Buffer A to Buffer TI), and Buffer TI (25 mM Tris‐HCl, 20 mM imidazole, pH 6.8). Bound proteins were eluted by boiling in 2× LDS loading buffer and analyzed by Western blotting using anti‐ETS1 antibodies to assess ETS1 ubiquitination levels.

### 4.12 Animal Experiments

Immunodeficient NSG mice (6‐8 weeks old) were subcutaneously injected with 5 × 10^6^ ccRCC cells. Tumor growth was monitored twice weekly using caliper measurements. When tumors reached 100–150 mm^3^, mice were randomized into treatment groups. cc‐885 was administered by oral gavage at 50 mg/kg daily (QD) or twice daily (BID). Control mice received vehicle (5% DMSO in PBS). Tumor volume was calculated using the following formula: (length × width^2^)/2. At the endpoint, the tumors were harvested for histological and molecular analyses. All animal experimental procedures were approved by the Institutional Animal Care and Use Committee of Sun Yat‐sen University (Approval Number: SYSU‐IACUC‐2025110037).

### 4.13 Graphical Abstract and Illustration Licensing

The graphical abstract and schematic illustrations (Figures 1F, 2J, 3B, 4E, 5A,G, 6L, and 7H) were created using BioRender.com. Publication rights for these figures have been secured under paid academic subscription licenses (Agreement Numbers: CZ292×1088, AA292×2TIR). All illustrations were adapted from BioRender templates, and the publication license has been obtained in accordance with BioRender's terms of use.

### 4.14 Statistical Analysis

All quantitative data were pre‐processed by normalization to designated internal controls (e.g., GAPDH for qPCR, total protein for Western blot). Data are presented as mean ± standard deviation (SD) unless otherwise stated in the figure legends. The sample size (n) for each experiment, representing biological replicates, is explicitly indicated in the respective figure legends. Specific statistical tests were chosen based on data distribution and experimental design: for comparisons between two groups, an unpaired, two‐tailed Student's t‐test was used; for multiple group comparisons, one‐way or two‐way analysis of variance (ANOVA) was performed, followed by Tukey's or Bonferroni's test for pairwise comparisons where applicable. The assumption of normality was assessed using the Shapiro‐Wilk test, and homogeneity of variances was verified using the Brown‐Forsythe test. Survival analysis was performed using the Kaplan‐Meier method, and curves were compared using the log‐rank test. The significance level (alpha) was set at *p* <0.05. All statistical analyses were performed using GraphPad Prism (version 9.5.1) or R software (version 4.2.2).

## Ethics Statement

The study was reviewed and approved by the ethics committee of The First Affiliated Hospital, Sun Yat‐sen University (Guangzhou, China).

## Consent

The content of the article, authorship, and ranking of all authors were carefully reviewed and agreed to be published.

## Conflicts of Interest

The authors declare no conflicts of interest.

## Supporting information




**Supporting File 1**: advs75521‐sup‐0001‐SuppMat.docx.

**Supporting File 2**: advs75521‐sup‐0002‐TableS1.xls.

## Data Availability

The raw data supporting the results of this study will be made available by the authors without undue reservation.
