# Supplementary material for: Molecular Glue cc‐885 Inhibits VHL‐Deficient Clear Cell Renal Cell Carcinoma via ETS1 Degradation
Source: Adv Sci (Weinh). 2026 May 6;13(42):e20237. doi: 10.1002/advs.202520237 (PMC13335456; doi:10.1002/advs.202520237)
Supplement: Supplementary file 1 — Supporting File 1: advs75521‐sup‐0001‐SuppMat.docx. [file ADVS-13-e20237-s001.docx]

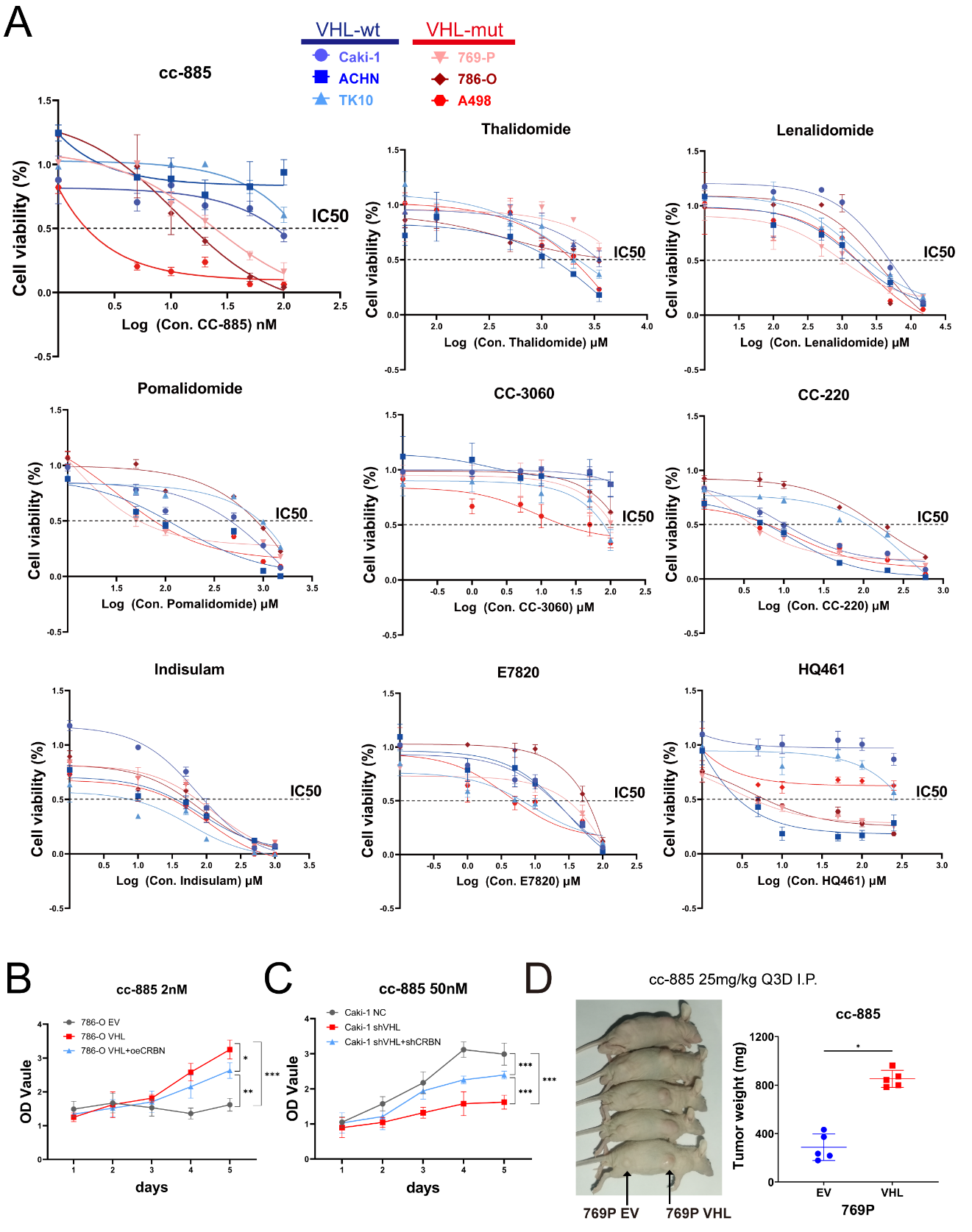


**Figure S1. cc-885 exhibits selective cytotoxicity in VHL-deficient ccRCC cells through CRBN-dependent mechanisms.**

**A**) Selectivity screening of molecular glues. Dose-response curves of cc-885 and eight additional molecular glue compounds in a panel of RCC cell lines. VHL-mutant cells (786-O, 769-P, A498) are shown in red; VHL-wild-type cells (Caki-1, ACHN, TK10) are shown in blue. Cell viability was measured after 48-hour treatment. Data points represent the mean ± SEM of n=5. The difference between the dose-response curves for VHL-mutant and VHL-wild-type cells was analyzed for each compound using an extra sum-of-squares F test.

**B**) Growth curves curves of 769-P (VHL-mutant) cells treated with 2 nM cc-885 or DMSO vehicle control over 120 hours. Cell viability was assessed at the indicated time points. Data are presented as mean ± SEM (n=5). Statistical significance at each time point was determined by two-way ANOVA with Šídák's multiple comparisons test. *P＜0.05, **P＜0.01, ***P＜0.001.

**C**) Growth curves of Caki-1 (VHL-wild-type) cells treated with 50 nM cc-885 or DMSO vehicle control over 120 hours. Cell viability was assessed at the indicated time points. Data are presented as mean ± SEM (n=5). Statistical significance at each time point was determined by two-way ANOVA with Šídák's multiple comparisons test. ***P＜0.001.

**D**) Immunodeficient NSG mice bearing 769-P (VHL-mutant) tumor xenografts were treated with vehicle or cc-885 (25 mg/kg) via intraperitoneal injection every three days. Each data point represents one horizontal lines indicate the mean ± SEM (n=5 mice per group). Statistical significance was determined by unpaired, two-tailed Student's t-test; *P < 0.001.


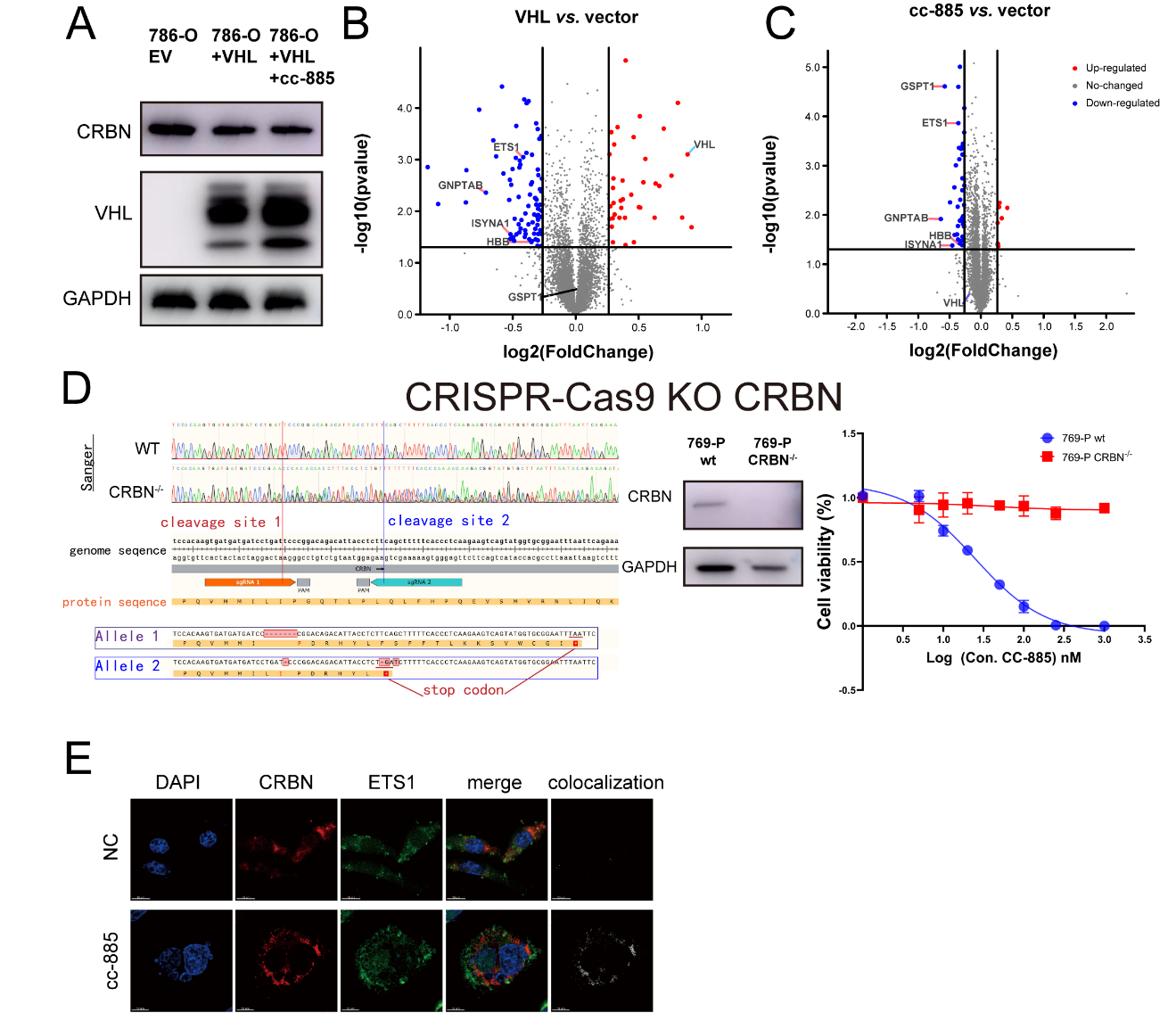


**Figure S2. Validation of CRBN-dependent ETS1 degradation and functional assays.**

**A)** Western blot analysis showing the expression levels of CRBN, VHL, and GAPDH proteins in 786-O cells treated with VHL and/or cc-885.
**B&C**) Volcano plots of TMT proteomics comparing (B) VHL-reconstituted vs. vector and (C) cc-885-treated vs. vector. Downregulated proteins (blue); upregulated proteins (red).

**D)** CRISPR-Cas9-mediated CRBN knockout in 769-P cells. ​**Left**: Genome sequencing of CRISPR cleavage sites. ​**Middle**: Western blot confirming CRBN knockout. ​**Right**: Dose-response curves showing reduced cc-885 sensitivity in CRBN-KO cells

**E**) Immunofluorescence staining of 786-O cells with DAPI (nuclei), CRBN (green), or ETS1 (red). cc-885 treatment enhances CRBN-ETS1 colocalization (white).


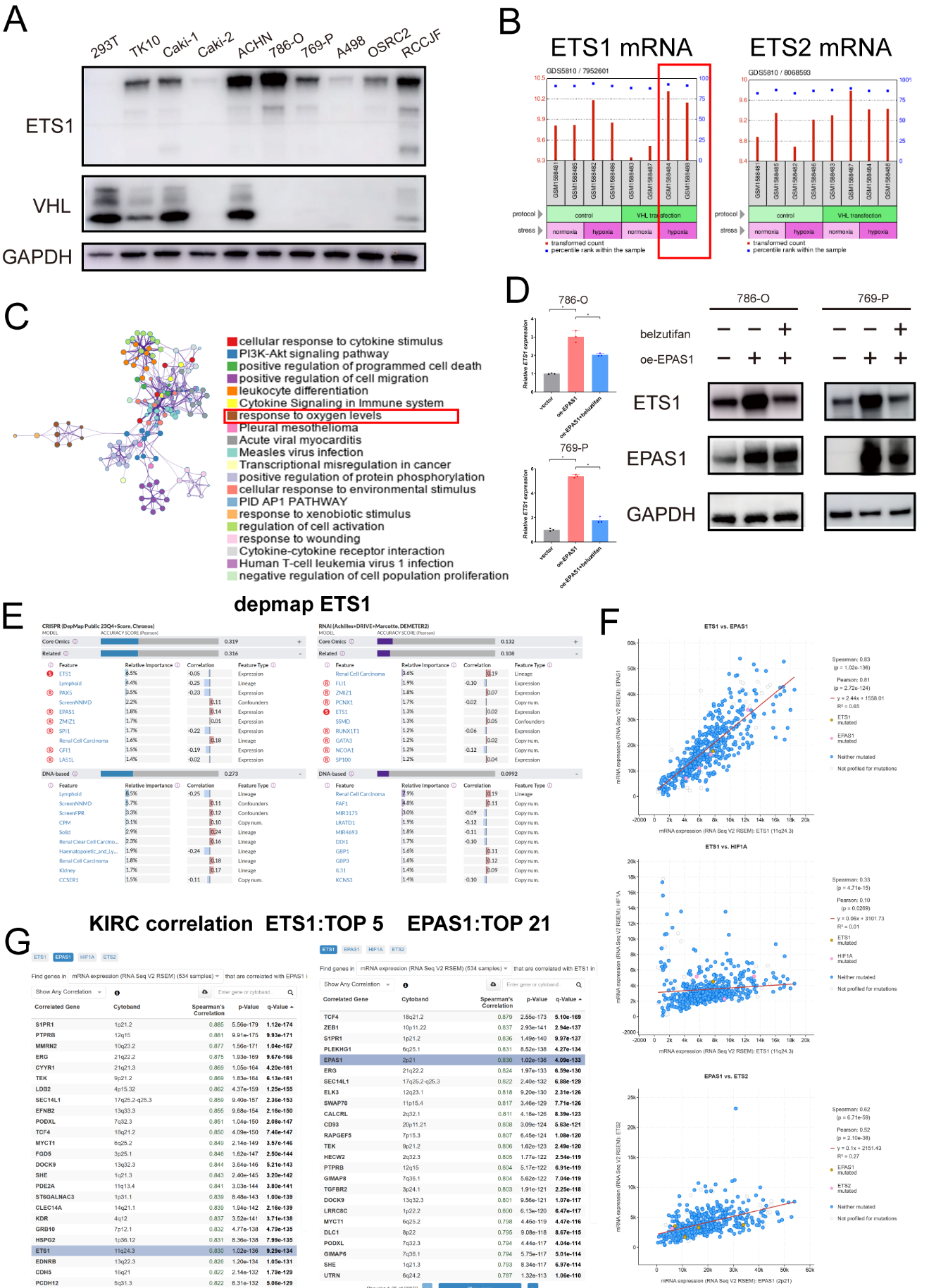


**Figure S3. Supplementary data validating ETS1 expression patterns, functional pathways, and EPAS1 co-regulation in ccRCC.
A)** Western blot analysis of ETS1, VHL, and GAPDH (loading control) across RCC cell lines (786-O, 769-P, A498) and non-malignant renal cells (293T, HK2)
**B)** mRNA expression levels of ETS1 and ETS2 in different samples are shown.

**C)** Gene Ontology (GO) enrichment analysis of ETS1-associated pathways.

**D)** (Left) RT-qPCR analysis of ETS1 mRNA in 786-O and 769-P cells with EPAS1 overexpression w/wo belzutifan (50 μM, 48 h). Data: mean ± SEM (n=4). One-way ANOVA with Tukey's test; *P<0.001 vs. Vector; (Right) Representative Western blots (n=3) of ETS1 and EPAS1 protein levels.

**E)** DepMap data analysis showing the relative importance, correlation, and feature type of various features associated with ETS1 in different cancer types.

**F)** Correlation scatterplot of ETS1 vs. EPAS1, ETS1 vs. HIF1A, ETS2 vs. EPAS1 mRNA expression in TCGA-KIRC cohort.

**G)** Correlation analysis of ETS1 and EPAS1 expression with other genes in KIRC (kidney renal clear cell carcinoma). The top 5 genes most correlated with ETS1 and the top 21 genes most correlated with EPAS1 are listed, along with their chromosomal location, Spearman's correlation coefficient, p-value, and q-value. The scatter plot on the right visualizes the correlation between these genes.

**Table S1**

| **Molecular glues** | **concentration range** | **E3 ligase** | **target proteins** |
| --- | --- | --- | --- |
| Thalidomide | 50-3500μM | CRL4^CRBN^ | IKZF1, IKZF3, ZNF692, ZNF276, SALL4, RNF166, ZBTB16, FAM83F, p63 |
| Lenalidomide | 10-15000μM | CRL4^CRBN^ | IKZF1, IKZF3, ZFP91, ZFP692, ZNF276, ZNF653, ZNF827, SALL4, RNF166, WIZ1, CK1α, FAM83F, RAB28 |
| Pomalidomide | 10-1500μM | CRL4^CRBN^ | IKZF1, IKZF3, ZFP91, ZFP692, ZNF276, ZNF653, ZNF827, SALL4, RNF166, GZF1, ZBTB39, ZNF98, WIZ1, ZBTB16, FAM83F, RAB28, DTWD1 |
| **CC-885** | 1-100**nM** | CRL4^CRBN^ | GSPT1, IKZF1, IKZF3, CDK4, PLK1, BNC2, BNIP3L |
| CC-220 | 1-600μM | CRL4^CRBN^ | IKZF1, IKZF3, ZFP91, ZNF98 |
| CC-3060 | 0.1-100μM | CRL4^CRBN^ | ZBTB16, IKZF1, ZFP91, ZNF276 |
| Indisulam | 1-1000μM | CRL4^DCAF15^ | RBM39、RBM23 |
| E7820 | 0.1-100μM | CRL4^DCAF15^ | RBM39 |
| HQ461 | 1-250μM | DDB1 | cyclin K、CDK12 |
